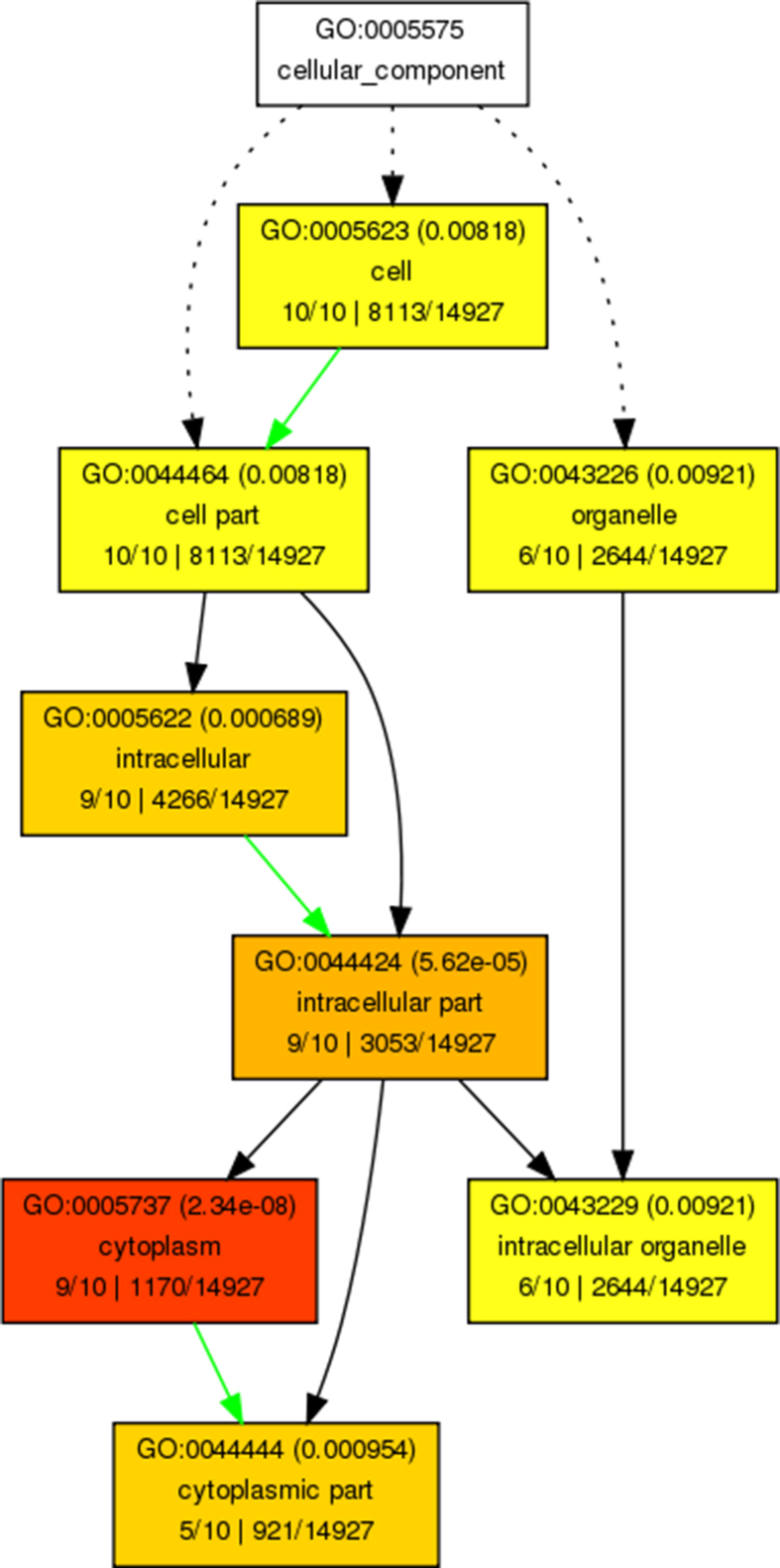# Exogenous interactome analysis of bovine viral diarrhea virus-host using network based-approach and identification of hub genes and important pathways involved in virus pathogenesis

**DOI:** 10.1016/j.bbrep.2024.101825

**Published:** 2024-09-16

**Authors:** Seyedeh Elham Rezatofighi

**Affiliations:** Biology Department, Faculty of Science, Shahid Chamran University of Ahvaz, Ahvaz, Iran

**Keywords:** BVDV, Cytoscape, GO, Hub gene, KEGG, Protein-protein interaction

## Abstract

Bovine viral diarrhea (BVD) is one of the most important diseases in livestock, caused by BVD virus (BVDV). During the pathogenesis of the virus, many interactions occur between host and viral proteins. Studying these interactions can help better understand the pathogenesis of the virus, identify putative functional proteins, and find new treatment and prevention strategies. To this aim, a BVDV-host protein-protein interaction (PPI) network map was constructed using Cytoscape and analyzed with cytoHubba, Kyoto Encyclopedia of Genes and Genomics (KEGG), Gene Ontology (GO), and Protein Analysis Through Evolutionary Relationships (PANTHER). N_pro_ with 125 connections had the greatest number of interactions with host proteins. *CD46*, *EEF*-2, and *TXN* genes were detected as hub genes using different ranking algorithms in cytoHubba. BVDV interactions with its host mainly focus on targeting translation, protein synthesis, and cellular metabolism pathways. Different classes of proteins including translational proteins, nucleic acid metabolism proteins, metabolite interconversion enzymes, and protein-modifying enzymes are affected by BVDV. These findings improve our understanding of the effects of the virus on the cell. Hub genes and key pathways identified in the present study can serve as targets for novel BVDV prevention or treatment strategies.

## Highlights

• BVDV-host interactions are mainly focused on targeting the translation, protein synthesis, and cellular metabolism pathways.
• N_pro_ of BVDV has the greatest number of interactions with host proteins.
• *CD46*, *EEF*-2, and *TXN* genes are hob genes.

## 1 Introduction

Bovine viral diarrhea (BVD) is a major disease in livestock, causing considerable economic losses to this industry. BVD is associated with gastrointestinal, respiratory, and reproductive disorders in cattle. The etiological agent of the disease is the BVD virus (BVDV), which belongs to the *Flaviviridae* family, *Pestivirus* genus. According to latest classification of *Pestivirus*, this genus has 11 species including pestivirus A (BVDV-1), pestivirus B (BVDV-2), pestivirus C (classical swine fever virus), pestivirus D (border disease virus), and pestiviruses E to K [1]. BVDV is a positive-sense single-stranded RNA virus that replicates in the cytoplasm. The RNA genome is approximately 12.3 kb in length and contains an open reading frame flanked by untranslated regions (UTRs). The ORF encodes a polyprotein precursor that is cleaved into 11 or 12 proteins in noncytopathic (ncp) or cytopathic (cp) virus biotypes, respectively. Mature viral proteins from N-terminus to C-terminus include N_pro_, capsid, E_rns_, E1, E2, p7, NS2-3, or NS2 and NS3, NS4A, NS4B, NS5A, and NS5B. BVDV-1 and BVDV-2 cause the same disease, but they are differ in the 5′-UTR sequences of their viral genomes [2].

Based on the cytopathic effects of BVDV on cell cultures, the virus is classified into two biotypes: cp and ncp [3]. The different features of BVDV biotypes result from differences in the processing of BVDV NS2-3. In cpBVDV infections, the NS2-3 protein is continuously cleaved by NS2 autoprotease activity to form NS3. However, in ncpBVDV infections, NS2-3 cleavage occurs during the early stages, and free NS3 is detected only up to 9 h post-infection. Therefore, the effects of NS2-3 and NS3 on cellular signaling appear to be different [4].

Viruses are intracellular parasites that need cells to replicate and survive. During various stages of viral infection, numerous interactions occur between host and viral proteins [5,6]. Investigating these interactions can improve our understanding of viral pathogenesis, help identify putative functional proteins, and facilitate the discovery of new treatment and prevention strategies. Host-viral protein-protein interactions (PPIs) have been studied through experimental approaches such as yeast two-hybrid, co-immunoprecipitation, tandem affinity purification-mass spectrometry, affinity chromatography, protein microarrays, protein-fragment complementation, and/or computational programs. Researchers have applied network biology approaches to construct disease networks that explore host-pathogen relationships and disease pathways [[6], [7], [8], [9], [10]]. Modeling virus-host interactome maps can help identify functionally important proteins [6], sub-networks, and potential hubs involved in the pathogenesis of specific diseases [7]. Researchers can then study these components using various bioinformatics methods. Viral proteins target bottlenecks, hubs, and rich clubs within cellular protein networks, thereby affecting cellular processes such as transcription, translation, cell signaling, cell trafficking, and cell cycle regulation [5,[11], [12], [13]].

The interaction of some BVDV proteins with host cell proteins has been previously investigated using experimental methods; however, there is no a single comprehensive network that encompasses all the research conducted on the BVDV interactome. This study aimed to combine previous works on BVDV-host interactions and construct a PPI network that encompasses all aspects of this topic. The goal of this study was to identify the proteins that significantly contribute to infection pathways, viral pathogenesis, and the virus's effects on host cells.

## 2 Material and methods

### 2.1 Data Collection

To perform this study, in the first step, all data related to the BVDV protein-protein interactions were collected. For this purpose, the advanced search option of PubMed was used. Multiple keywords related to interactions between BVDV and host proteins or virus proteins were applied. The search yielded 2690 research studies in which the virus was studied in different aspects including pathogenesis, epidemiology, and others. After a careful review of the articles, 24 papers contained the PPI data (Table 1) [2,4,[14], [15], [16], [17], [18], [19], [20], [21], [22], [23], [24], [25], [26], [27], [28], [29], [30], [31], [32], [33], [34], [35]]. In these articles, various PPI detection methods such as yeast two-hybrid, immunoprecipitation, co-immunoprecipitation and mass spectrometry were carried out. Besides the data, other PPI database including STRING Viruses [36], VirusMINT [37], IntAct [38], VirHostNet [39], and VirusMentha [40] were investigated. The information related to every single interaction of BVDV-1 or BVDV-2 with host were gathered. Because the BVDV-1 and BVDV-2 genes are identical and differ only in the 5′-UTR region, one network was drawn for both. In the context of virus–host interactions, protein-protein interfaces can be classified as exogenous or endogenous. Host-host or virus-virus PPIs are classified as endogenous interfaces, while interactions between different proteomes including virus–host PPIs are grouped as exogenous interfaces [41,42]. In the present study, host-host endogenous interfaces were excluded. All data gathered were convert to a unique format in order to construct a network in Cytoscape, because all data must be in the same format. Different identifiers including protein name, Uniprot, EMBL/GenBank, and others were converted to the same format, which here was the name gene. This work was done by an online Uniprot ID Mapping (http://www.uniprot.org/mapping/) [43]. Then, the data gathered from all resources were merged and duplicates were removed.

**Table 1:** List of studies which reported the protein-protein interaction data of BVDV-host.

No	Paper	No. of interactions	References
1	Weiskircher et al., 2009	2	2
2	Yamane et al., 2009	2	4
3	Lazar et al., 2003	1	14
4	Hilton et al., 2003	1	15
5	Zahoor et al., 2010	1	16
6	Gong et al., 2020	1	17
7	Johnson et al., 2001	1	18
8	Leveringhaus et al., 2020	1	19
9	Mu et al., 2021	1	20
10	Branza-Nichita et al., 2004	1	21
11	Fu et al., 2014	4	22
12	Darweesh et al., 2018	1	23
13	Schaut et al., 2015	1	24
14	Xu et al., 1997	4	25
15	Branza-Nichita et al., 2001	1	26
16	Maurer et al., 2004	1	27
17	Lackner et al., 2005	1	28
18	Chen et al., 2007	1	29
19	Dubrau et al., 2017	2	30
20	Mohamed et al., 2014	1	31
21	Wang et al., 2011	7	32
22	Jefferson et al., 2014	89	33
23	Ammari et al., 2017	72	34
24	Gladue et al., 2014	31	35

### 2.2 PPI network Construction and analysis

The latest version of Cytoscape (v3.9.0) [44] was used to construct the BVDV-host PPI network. Cytoscape is a user-friendly platform which help to construct, visualize and analyze of the biomolecular interaction networks. This platform has various tools and plugins for understanding and exploration of the various aspects of the network. Cytoscape Analyzer tool was used to define the topological characters of the network including diameter, radius, average number of neighbors, density, clustering coefficient, and so on.

### 2.3 Hub genes and modules analysis

CytoHubba (v0.1) [45], a plugin in Cytoscape was used to identify the hub genes. Top ten genes were selected with five algorithms in cytoHubba including MCC, Density of Maximum Neighborhood Component (DMNC), Edge Percolated Component (EPC), Radiality centrality, and Stress centrality. Different clustering algorithms including MCODE (v2.0.0) [46], SCODE (v1.0.4) [47], CytoCluster (v2.1.0) [48], and ClusterViz (v1.0.3) [49] were applied to construct modules and sub-networks. These are plugins in Cytoscape software for clustering nodes based on different algorithms. These plugins can find densely connected nodes [50], important protein complexes [2] and valuable functional motifs. Default parameters were applied for all plugins to construct sub-networks.

### 2.4 Functional enrichment analysis

Gene enrichment analysis was performed to evaluate the biological pathways of proteins that might be involved in the development and pathogenesis of BVDV. KEGG pathway enrichment analysis was performed using free web based search engine DAVID program (v6.8) with *P* < 0.05 threshold (https://david.ncifcrf.gov/) [51]. GO enrichment analysis was done using AgriGO (v2.0) to evaluate the CC, MF, and BP of involving host proteins. Interacting host proteins were analyzed with Singular Enrichment Analysis (SEA) tool. Gene names were converted to UniProtKB format because AgriGO allowed ID types including *Bos taurus* NCBI-GI, ENSEMBL ID, Bovine genome locus (Bovine Genome Database), GenBank ID, DDBJ ID, EMBL ID, UniProt ID, RefSeq Peptide ID, PDB ID, and Bovine Affymetrix Genome Array. The AgriGO can be found at (http://bioinfo.cau.edu.cn/agriGO/) [52].

BVDV-interacting host proteins were entered into the PANTHER classification program (v16.0). The PANTHER system classifies proteins and their genes according to family and subfamily, molecular function, biological process, and pathway. The PANTHER is online freely program and can be found at (http://www.pantherdb.org/) [53].

## 3 Results

### 3.1 The BVDV-host protein interaction network

After extracting data from literature and databases, the PPI network was constructed using Cytoscape. In total, 224 interactions were confirmed by experiment-derived data from previous studies. These interactions involved viral proteins including N_pro_ (99 interactions), NS3 (77 interactions), E2 (34 interactions), E_rns_ (5 interactions), NS5A (4 interactions), NS4A (one interaction), C (one interaction), E1 (one interaction), NS4B (one interaction), and E1 (one interaction) with host or viral proteins. The total number of interactions derived from previous studies and data bases was 561; however, after removing duplicates, 279 nodes and 436 interactions were identified (Fig. 1). Among the 279 nodes, 12 were viral proteins, while remaining were host proteins. N_pro_ with 125 connections, had the greatest number of interactions with host proteins, followed by NS2-3, NS3, and E2, with 94, 87, and 65 associations, respectively. Fig. 2 shows the number of interactions between each BVDV-protein and host proteins. The PPI network was analyzed using the network analysis tool, and important statistical features are presented in Table 2.

**Fig. 1:**
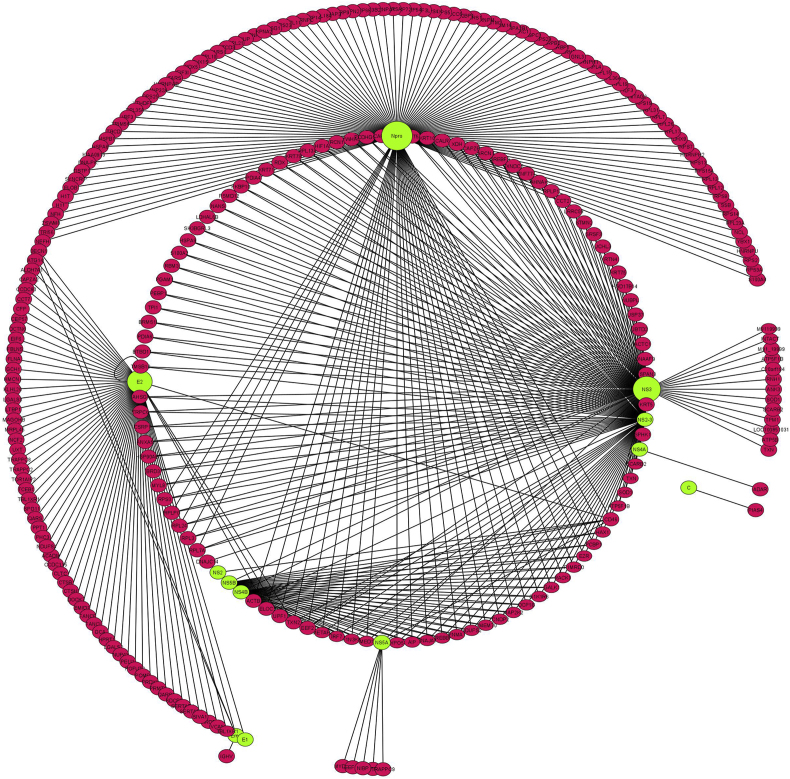
BVDV-Host protein interaction network constructed and visualized using Cytoscape. Red nodes represent host genes, green nodes represent virus genes, and size of nodes represent the number of genes which interacted with the node. Larger nodes have more interactions with the genes. (For interpretation of the references to colour in this figure legend, the reader is referred to the Web version of this article.)

**Fig. 2:**
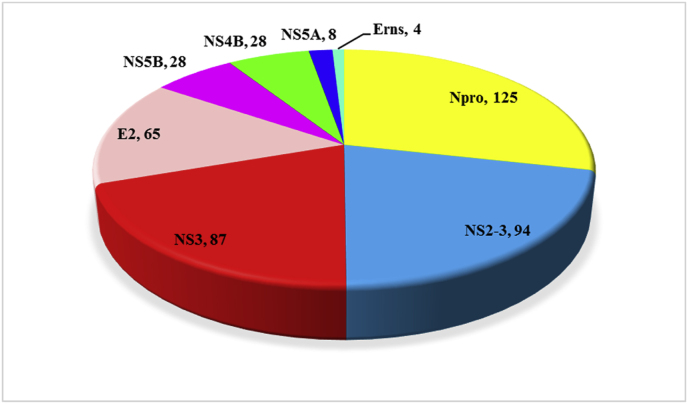
A pie chart representing the number of BVDV genes interaction with host genes. In each section, the name of the virus gene and the number of interactions with the host genes are represented.

**Table 2:** Topological characters of BVDV-host network.

Number of nodes	279
Number of edges	436
Avg. number of neighbors	3.118
Network diameter	8
Characteristics path length	3.506
Clustering coefficient	0.012
Network density	0.006

### 3.2 Screening of hub genes and modules analysis

Different ranked algorithms in CytoHubba were used to identify host hub genes. The ranks of top ten hub genes selected by each feature are shown in Table 3. *CD46* and *EEF2* genes were identified by all five algorithms, also *TXN2* was detected by four of five ranked methods in cytoHubba.

**Table 3:** Top ten hub genes detected using different topological algorithms in cytoHubba.

Algorithm	MCC	DMNC	MNC	Radiality	Stress
1	CD46^a^	CD46^a^	CD46^a^	CD46^a^	CD46^a^
2	EEF2^a^	EEF2^a^	EEF2^a^	TXN2^b^	TXN2^b^
3	TXN2^b^	ACTB	TXN2^b^	EEF2^a^	EEF2^a^
4	ACTB	HAX1	TNEM70	RPLPO	RPLPO
5	HAX1	PCBP1	CNDP2	RPL7A	RPL7A
6	PCBP1	EZR	PNMA1	RPL24	RPL24
7	EZR	ACTG1	ACTG1	RPL3	RPL3
8	ACTG1	RACK1	TMED 10	RPS3	RPS3
9	RACK1	GALK1	AIP	ACTB	ELOC
10	GALK1	G1K1R6	G1K1R6	HAX1	UPF1

MCC: Maximal Clique Centrality; DMNC: Density of Maximum Neighborhood Component; EPC: Edge Percolated Component.

a The hub genes identified by all algorithms in cytoHubba.

b The hub genes identified by four ranked algorithms in cytoHubba.

**Fig. 3:**
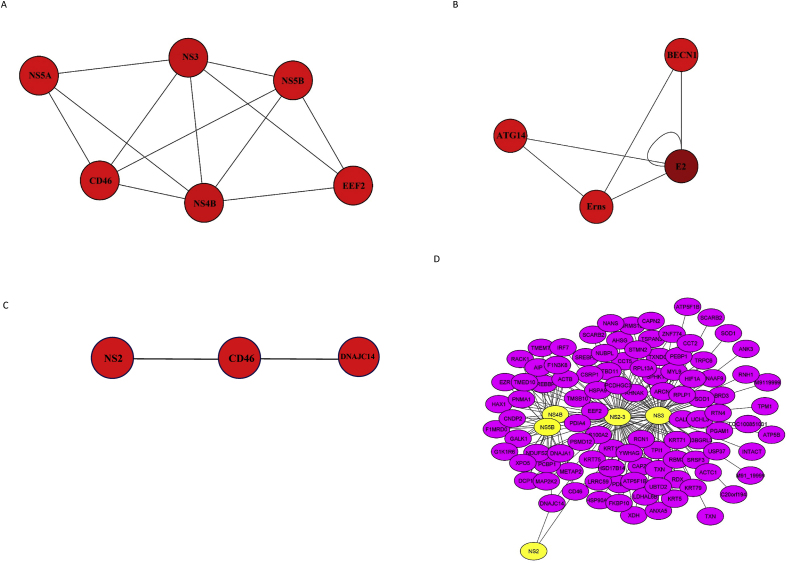

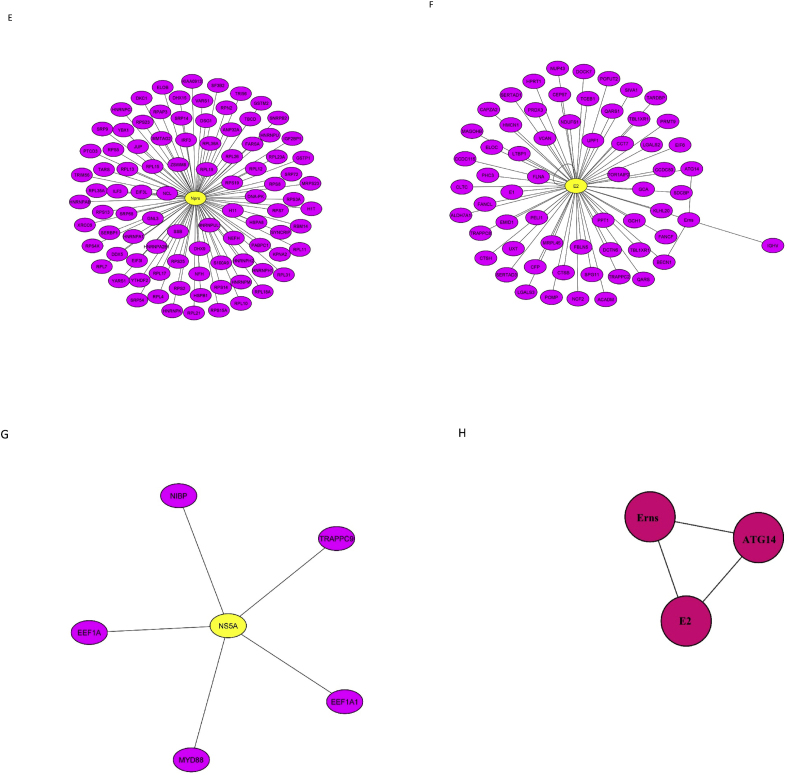
Key moddules for the whole network by MCODE, CytoCluster, ClusterViz, and SCODE. (A and B) Sub-clusters constructed using the MCODE plugin. (C) Sub-cluster identified using CytoCluster. (D, E, F, and G) Sub-clusters constructed using the ClusterVis plugin. Virus genes are showon in red, while host genes are presented in purple. (H) Modules constructed using SCODE. (For interpretation of the references to colour in this figure legend, the reader is referred to the Web version of this article.)

Different sub-networks were constructed using various clustering algorithms to find important protein complexes and functionally modules in BVDV-host protein interaction network. Fig. 3 shows multiple clusters made for the whole network by Molecular Complex Detection (MCODE), CytoCluster, ClusterViz, and Supervised Complex Detection (SCODE). Two sub-cluster were constructed by MCODE. Sub-cluster 1 had 6 nodes in which 4 nodes belonged to viral proteins and 2 belonged to host (Fig. 3A). Sub-cluster 2 consisted of 4 nodes, two of which belonged to host genes (Fig. 3B). In the cluster made by CytoCluster, there were 3 nodes among which NS2 belonged to viral proteins while CD46 and DNAJC14 were bovine proteins (Fig. 3C). According to cluster analysis performed by ClusterVis, four smaller sub-networks were constructed. First sub-network (Fig. 3D) contained 106 nodes among which NS2, NS3, NS2-3, NS4B, and NS5B were viral proteins and the others belonged to host proteins. Second sub-network consisted of 92 nodes, in which N_pro_ was viral protein (Fig. 3E). Third sub-network had 63 nodes among which E2 was viral protein (Fig. 3F). Forth sub-network was consisted of viral protein NS5A and five host proteins (Fig. 3G). Cluster constructed by SCODE had 3 nodes of E_rns_ and E2 (viral proteins), and ATG14 (host protein).

### 3.3 KEGG and GO enrichment analysis

**Fig. 4:**
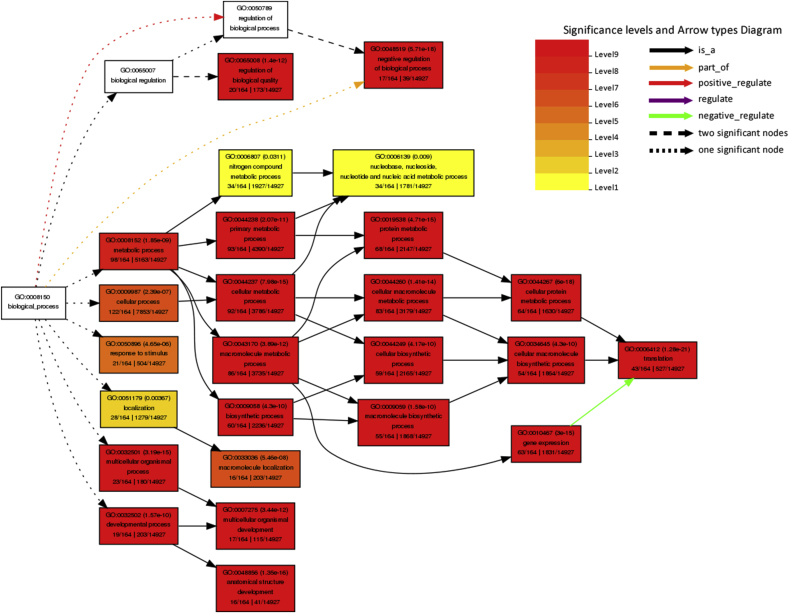

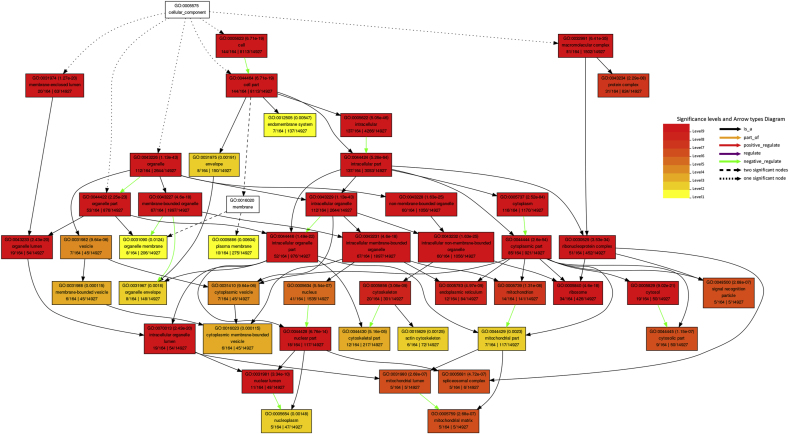

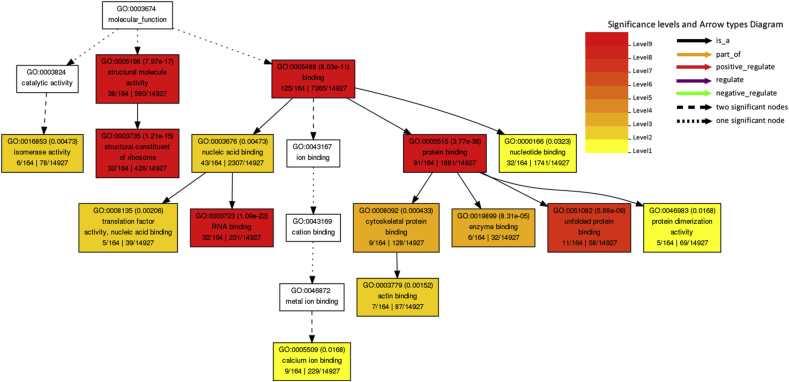
The Gene Ontology (GO) enrichment analysis using AgriGO**.** (A) GO terms biological process (BP). (B) GO terms cellular compenets (CC). (C) GO terms molecular functions (MF). GO term codes are shown in braces.

Kyoto Encyclopedia of Genes and Genomics (KEGG) pathway analysis was performed using the Database for Annotation, Visualization, and Integrated Discovery (DAVID) database for all host proteins interacted with BVDV and specifically top ten hub genes ranked by CytoHubba based on Maximal Clique Centrality (MCC) algorithm. Table 4 shows the KEGG pathway analysis for all BVDV-interacting host proteins. The interacting proteins were highly enriched in ribosome, spliceosome, Influenza A, protein processing in endoplasmic reticulum, proteoglycans in cancer, measles, RNA transport, and protein export while KEGG pathway analysis of top 10 hub genes revealed that these genes were involved in leukocyte transendothelial migration. The Gene Ontology (GO) function analysis was carried out using AgriGO. The results showed that the proteins were significantly involved in biological processes such as metabolic (98 genes), cellular (122 genes), localization (28 genes), response to stimulus (21 genes), biosynthetic (60 genes), gene expression (63 genes), translation (43 genes), multicellular organismal (23 genes), developmental (19 genes), etc processes (Fig. 4A). For GO cellular component analysis, the proteins were mainly associated with cytoplasm (116 genes), organelle (112 genes), nucleus part (41 genes), ribosome (34 genes), cytoskeleton (20 genes), mitochondrion (14 genes), and endoplasmic reticulum (12 genes) (Fig. 4B). The proteins significantly enriched in molecular functions including binding (125 genes), protein binding (91 genes), structural molecule activity (38 genes), structural constitute of ribosome (32 genes), RNA binding (32 genes), translation factor activity (5 genes), etc (Fig. 4C). The GO cellular component (CC), and molecular function (MF) of the top hub genes ranked by MCC algorithm are shown in Supplementary Fig. 1. The GO biological process (BP) of hub genes was not significant (*P* > 0.05).

**Table 4:** Kyoto encyclopedia of Genes and Genomes (KEGG) pathway enrichment analysis of host genes interacted with BVDV genes.

Term	Count (%)	*P*-Value	Benjamini
Ribosome	35 (14.5)	8.7E-30	1.4E-27
Spliceosome	13 (5.4)	5.7E-6	4.7E-4
Influenza A	10 (4.1)	5.7E-3	2.4E-1
Protein processing in endoplasmic reticulum	9 (3.7)	1.5E-2	5.1E-1
Proteoglycans in cancer	8 (3.3)	9.3E-2	1.0E0
Measles	7 (2.9)	5.1E-2	1.0E0
RNA transport	7 (2.9)	8.3E-2	1.0E0
Protein export	5 (2.1)	1.0E-3	5.5E-2

### 3.4 PANTHER classification of proteins

**Fig. 5:**
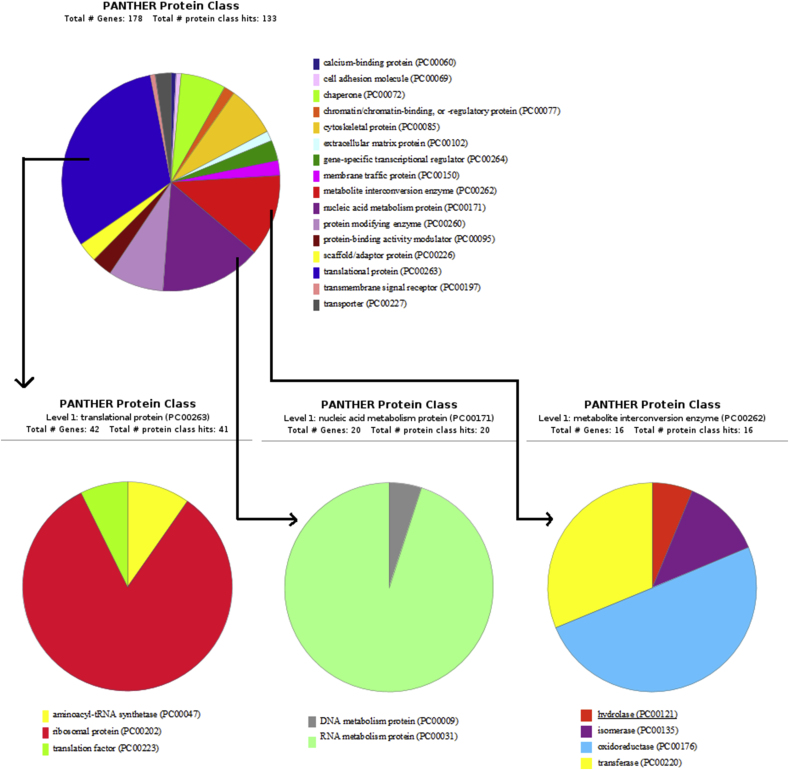
Panther pie-chart of host interacting proteins classification. The three main GO terms were subclassified to child categories (pie charts at the bottom of the image). GO term codes are shown in braces.

The host proteins that interact with BVDV were classified using the Protein Analysis Through Evolutionary Relationships (PANTHER) Classification System. According to the protein classes of host targeted genes, translational proteins, nucleic acid metabolism proteins, metabolite interconversion enzymes, and protein modifying enzymes were the most likely candidates as targets of viral proteins (Fig. 5). Some of these proteins involve in various biological categories as cellular proteins often play several roles in several pathways.

## 4 Discussion

We constructed a comprehensive BVDV-host protein interaction map to describe the interactome between BVDV and host proteins, aiming to better understand the pathogenicity of this virus and identify potential drug targets for the treating of BVDV infection. Although BVDV, like other RNA viruses, produces few proteins, these proteins often interact with multiple host proteins to create favorable conditions for the virus's survival and replication [14]. Identifying of host proteins targeted by viral proteins and investigating under both of their functions in normal and infectious conditions can help us to uncover the key processes required for infection [14]. In this context, network biology can be a useful tool for studying host-pathogen relationships at the molecular level. Various researchers have used network biology to discover key viral proteins and their targets in infection processes [6,7,9]. In the present study, all previous small-scale, large-scale, and computational investigations on BVDV-host interactions were integrated to detect highly BVDV-associated host proteins and their roles in viral pathogenesis.

In cellular network analysis, proteins with a wide range of connecting partners are called “hubs” [54]. After constructing the PPI network, 10 hub genes were identified using five different ranking methods. The *CD46*, *EEF-2*, and *TXN* genes were detected by at least four out of the five ranking methods in cytoHubba. *CD46*, the most prominent identified hub gene, is a ubiquitously expressed “multitasker,” found on all nucleated cells, protecting them from autologous damage by the complement system [55,56]. *CD46* regulates both adaptive immunity and the complement system [55]. It has been identified as interacting with other pathogens, such as like the measles virus [55], human herpesvirus 6 [56], group B adenoviruses [57], and pathogenic Neisseria [58]. *TXN*-2, also known as *Trx*-2, is involved in various cellular processes, including the regulation of ROS production, apoptosis, cell growth, and the modulation of transcription factors [59]. Researchers have reported that *TXN*-2 plays a role in the life cycle of the classical swine fever virus (CSFV) and BVDV [59,60]. *TXN-2* inhibits CSFV replication, and in the case of BVDV, it is downregulated by the virus during early infection. *EEF-2* encodes eukaryotic elongation factor 2, which is essential for protein synthesis [61].

KEGG pathway analysis revealed that BVDV proteins primarily target biological pathways involved in translation and protein synthesis, including ribosome, spliceosome, protein processing in the endoplasmic reticulum, and RNA transport. BVDV interacts with ribosomal complexes to ensure that viral RNAs are translated and can produce substantial amounts of viral proteins even under the strict conditions imposed by the host shutoff mechanism, where cellular metabolism is severely limited [14,62]. As found in the present study and reported by other researchers, BVDV manipulates biological processes such as cellular metabolism, gene expression, translation, cell development and differentiation [[62], [63], [64]]. The cellular proteins and pathways targeted by BVDV proteins, including RNA binding, protein binding, spliceosome, translation, and biosynthesis, are also involved in the pathogenesis of other viruses, indicating common strategies among these viruses [54,63,65]. According to KEGG analysis, the enriched gene set is found to be involved in other infectious pathways such as Influenza A virus and measles infections.

GO enrichment analysis revealed that most interacting proteins function in various biological processes, including cellular protein metabolism, cellular macromolecular biosynthesis, gene expression, and translation. These proteins are primarily found in the nucleus, ribosome, cytoskeleton, mitochondrion, and endoplasmic reticulum. Most of the host-interacting proteins have binding molecular functions, such as protein binding, RNA binding, translation factor activity-nucleic acid binding, and cytoskeletal protein binding. Some of these proteins also serve as structural constituents of the ribosome. The PANTHER, GO, and KEGG evidence highlights the widespread effects of BVDV on cellular processes, particularly in the translation and synthesis of proteins, as reported in other studies [62,63].

## 5 Conclusion

Although BVDV encodes few proteins (like other RNA viruses), it can effectively affect multiple host pathways. The BVDV-host PPI network map, constructed based on small-scale, large-scale, and computational studies, revealed that viral proteins target various protein classes, including translational proteins, nucleic acid metabolism proteins, metabolite interconversion enzymes, and protein-modifying enzymes. *CD46*, *EEF-2*, and *TXN* were identified as hub genes using different ranking algorithms. The present study shows that BVDV interactions with its host primarily focus on targeting translation, protein synthesis, and cellular metabolic pathways. Further studies are needed to explore the effects of these proteins on cellular pathways and the molecular mechanisms of BVDV infection.

## Funding

No funding.

## Compliance with ethical standards

Not applicable.

## Availability of data and materials

The datasets used and/or analyzed during the current study are available through the paper and additional files.

## CRediT authorship contribution statement

**Seyedeh Elham Rezatofighi:** Writing – original draft, Visualization, Validation, Supervision, Project administration, Methodology, Investigation, Formal analysis, Data curation.

## Declaration of competing interest

The authors declare no conflict of interests.

## Figures and Tables

**figs1:**
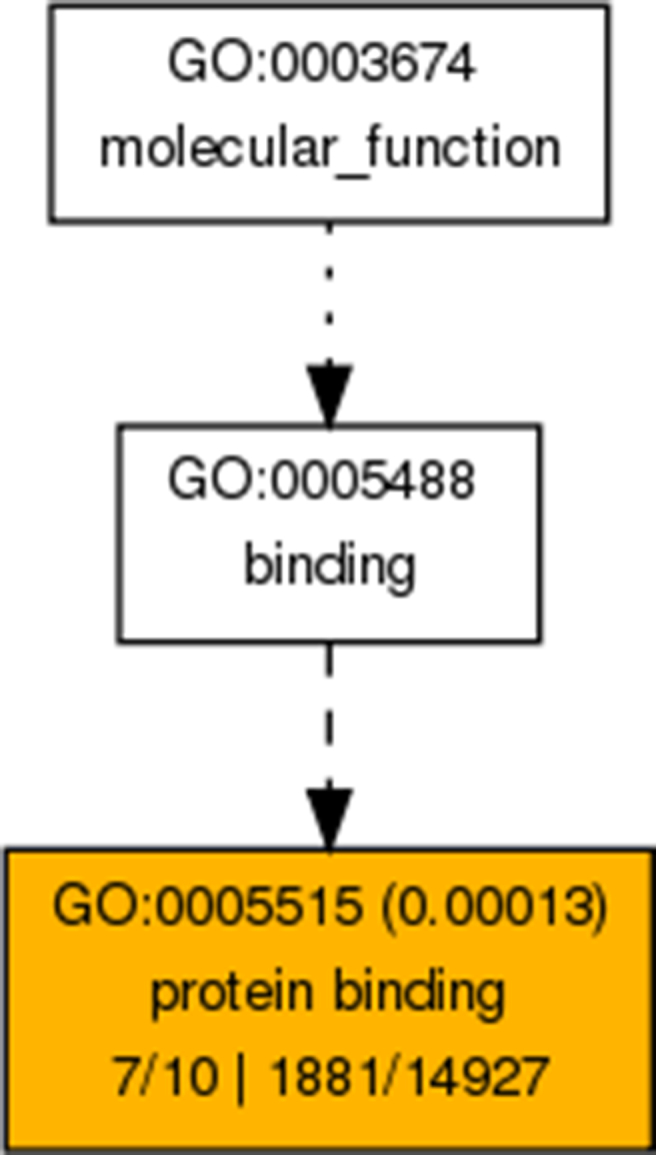


**figs2:**