# Supplementary material for: Exogenous interactome analysis of bovine viral diarrhea virus-host using network based-approach and identification of hub genes and important pathways involved in virus pathogenesis
Source: Biochem Biophys Rep. 2024 Sep 16;40:101825. doi: 10.1016/j.bbrep.2024.101825 (PMC11421936; doi:10.1016/j.bbrep.2024.101825)
Supplement: Multimedia component 1 [file mmc1.docx]

**Supplementary files**

**Supplementary Figure 1.** The Gene Ontology (GO) enrichment analysis of hub genes ranked by MCC algorithm (a) GO terms cellular compenets (CC). (b) GO terms molecular functions (MF). GO term codes show in braces. GO term codes are shown in braces.

**Supplementary Table 1.** Kyoto encyclopedia of Genes and Genomes (KEGG) pathway enrichment analysis of hot genes interacted with BVDV genes.

**Supplementary data file.** Protein-protein interactions (PPI) between BVDV and host proteins presented in the study
