# Supplementary material for: Exogenous interactome analysis of bovine viral diarrhea virus-host using network based-approach and identification of hub genes and important pathways involved in virus pathogenesis
Source: Biochem Biophys Rep. 2024 Sep 16;40:101825. doi: 10.1016/j.bbrep.2024.101825 (PMC11421936; doi:10.1016/j.bbrep.2024.101825)
Supplement: Multimedia component 2 [file mmc2.docx]

Supplementary Table 1.

| **Term** | **Count (%)** | ***P*-Value** | **Benjamini** |
| --- | --- | --- | --- |
| Leukocyte transendothelial migration | 3 (30) | 4.7E-3 | 1E-1 |
| Oxytocin signaling pathway | 3 (30) | 7.2E-3 | 1.0E-1 |
| Proteoglycans in cancer | 3 (30) | 1.4E-2 | 1.1E-1 |
| Regulation of actin cytoskeleton | 3 (30) | 1.5E-2 | 1.1E-1 |
